# Effectiveness of Compassion Training on Stress and Anxiety: A Pre-Experimental Study on Nursing Students

**DOI:** 10.3390/nursrep14040268

**Published:** 2024-11-27

**Authors:** Andrés Gutiérrez-Carmona, Marta González-Pérez, María Dolores Ruiz-Fernández, Angela María Ortega-Galán, Diego Henríquez

**Affiliations:** 1 Department of Nursing, University of Antofagasta, Antofagasta 1240000, Chile; 2 Department of Nursing, University of Huelva, 21004 Huelva, Spain; marta.gonzalez210@alu.uhu.es (M.G.-P.); angela.ortega@denf.uhu.es (A.M.O.-G.); 3 Department of Nursing, Physiotherapy and Medicine, University of Almería, 04120 Almería, Spain; mrf757@ual.es; 4 Facultad de Ciencias de la Salud, Universidad Autónoma de Chile, Temuco 4780000, Chile; 5 School of Psychology and Philosophy, University of Tarapacá, Arica 1000000, Chile; dthenriquezh@academicos.uta.cl

**Keywords:** nursing, student, compassion, stress, anxiety, intervention

## Abstract

Background: Stress and anxiety are common problems among nursing students, affecting their mental health and academic performance. Compassion training has been shown to be effective in reducing these states. Objectives: This study evaluated the effects of a compassion-based course on perceived stress and anxiety (state and trait) among nursing students at a state university in Chile. Methods: A pre-experimental design was implemented with 45 second-year students, who completed evaluations before and after the intervention. The course included 10 sessions involving compassion meditation, mindfulness, self-compassion exercises, and autonomous practice. Results: The results showed significant reductions in and a high effect size regarding perceived stress (Cohen’s *d* = 0.83) and state anxiety (Cohen’s *d* = 0.74), while trait anxiety showed a moderate reduction (Cohen’s *d* = 0.34). Mediation analysis revealed that increases in self-compassion mediated the relationship between autonomous practice time and reductions in stress and trait anxiety. Conclusions: These findings suggest that compassion training is an effective strategy for reducing stress and anxiety in nursing students, improving their emotional regulation and preparation for professional practice. Therefore, it is necessary to include this approach in students’ curricular programs.

## 1. Introduction

Stress and anxiety are prevalent problems among both healthcare professionals and students in disciplines such as nursing, affecting their mental well-being and performance in clinical and academic settings [1,2,3]. For professionals, continuous exposure to emotionally demanding situations, coupled with a high workload, a lack of resources, and the risk of making mistakes, has been shown to negatively impact their mental health [2,4]. This not only compromises the well-being of professionals but can also impact the quality of care provided to patients [4].

Anxiety is defined both as a transitory state and a lasting trait. State anxiety (A-State) is described as a temporary emotional response characterized by conscious feelings of tension and apprehension accompanied by increased autonomic nervous system activation [5]. In contrast, trait anxiety (A-Trait) refers to a relatively stable individual disposition toward anxiety, that is, differences between individuals in terms of their tendency to react with heightened A-State intensity to situations perceived as threatening [5].

To define stress in this study, we adopted the conceptualization proposed by the authors of the Chilean adaptation of the Global Perceived Stress Scale [6]. According to this definition, stress is based on the subjective assessment each person makes of a stressful event, which must meet certain criteria: it must be perceived as a threatening or demanding situation, and the resources available to cope with it must be considered insufficient. In this context, stress is understood as the result of a cognitive evaluation that mediates the individual’s emotional response to the event in question.

While stress and anxiety have been directly linked to professional practice in nursing [2], research suggests that these states begin to manifest as early as the formative stage [1]. Stress and anxiety levels have been reported to be significantly higher in nursing students than in students in other disciplines [7], with a high prevalence of psycho-emotional problems [3,8] and academic burnout syndrome [9]. These findings underscore the need to create environments and formative strategies that allow students to develop skills to manage stress and anxiety, both in their educational stage and in their future careers.

In this context, compassion training has emerged as a potentially effective approach to reducing stress and anxiety in both students and healthcare professionals [1,2]. Additionally, incorporating compassion as part of healthcare training could contribute to the creation of more humanized environments in both educational and clinical settings [1,2,10].

Compassion is defined as the capacity to be sensitive to one’s own and others’ suffering, accompanied by the motivation to prevent and alleviate it [11,12]. For compassion to manifest fully and virtuously, it must flow in three directions: towards oneself (self-compassion), towards others, and through the compassion we receive from others [11,12].

Scientific evidence suggests that compassion is a skill that can be developed and strengthened through practice [11,13]. Recent research has shown that compassion training not only induces functional changes but also anatomical modifications in the brain [14]. The continued practice of showing compassion is associated with increased activation and connectivity in key brain areas for empathy and emotional regulation, such as the prefrontal cortex and limbic system [14].

Compassion-training programs use a variety of techniques rooted in both ancient traditions and contemporary psychology [11]. Among the most prominent are compassion meditation (Metta Bhavana), which emphasizes cultivating feelings of kindness and love towards oneself and others, extending this benevolence even to those with whom one has disagreements; mindfulness, which promotes awareness of the present moment and facilitates more compassionate attitudes by heightening awareness of one’s own and others’ suffering; and self-compassion exercises, which encourage a show of kindness towards oneself in times of adversity rather than self-criticism [15]. In addition, guided imagery, gratitude journaling, and active listening and empathy are key to strengthening compassion [11,16].

A systematic review of compassion-based intervention programs applied to various population groups found a moderate effect on improving life satisfaction as well as reducing anxiety- and stress-related suffering [17]. Congruently, other systematic reviews have reported that compassion training not only increases levels of self-compassion but also reduces psychopathology, with effect sizes ranging from moderate to large [18]. Meanwhile, a recent meta-analysis by Alcaraz-Cordoba [15] involving health professionals revealed that compassion-training programs generate significant changes in self-compassion levels, albeit with small effect sizes. On the other hand, a study by Neff [16] confirmed that participation in such programs increases levels of self-compassion substantially, with notable effect sizes. Likewise, a potential mediating effect of self-compassion on the relationship between compassion training and different variables has been observed [19,20].

Given nursing students’ challenging formative context and the work context they will face as professionals, it is highly relevant to incorporate and evaluate evidence-based professional subjects to address stress and anxiety as a basis for effective supportive relationships and humanized care. There is also a growing need for educational and prevention approaches to address the root causes of mental health problems faced by university students [13]. Despite these advances, there are few studies exploring compassion training in relation to nursing students, let alone the formal inclusion of this type of training in curricular plans. In addition, no undergraduate nursing programs have been identified that evaluate the impact of self-compassion training on student stress and anxiety.

Therefore, the objectives of this research are (a) to evaluate the effect of a subject based on compassion training on stress and anxiety levels (trait and state) and (b) evaluate the mediating effect of changes in self-compassion on the relationship between autonomous practice time and its effect on stress and anxiety (trait and state).

## 2. Materials and Methods

### 2.1. Design and Participants

In a quantitative study with a pre-experimental design, a subject focused on compassion training in a nursing degree program was evaluated. The sample consisted of 59 second-year students enrolled in the nursing degree program at the University of Antofagasta (Chile). The inclusion criteria were as follows: the subjects had to be enrolled in a course on the subject ‘Human Development’ and had to have agreed to participate in this study by signing the informed consent form. Students who did not want to participate and did not sign the informed consent form, as well as those who did not complete the questionnaires correctly or forgot their registration codes during the post-test, were excluded. 

Of the 59 students, 53 participated in the pre-test, and 50 participated in the post-test. The final study sample included 45 nursing students, as 5 participants who forgot their identification codes on the post-test were excluded. The pretest was conducted in August 2023, before the intervention, and the posttest was conducted in December 2023, at the end of the intervention.

### 2.2. Intervention

The intervention was restructured into 10 face-to-face sessions lasting approximately 135 min each, complemented by weekly autonomous non-face-to-face exercises. Techniques employed included (a) compassion meditation (Metta Bhavana), which focused on cultivating feelings of kindness and love towards oneself and others, progressively extending this benevolence even towards people with whom there were conflicts; (b) mindfulness, which promoted full awareness of the present moment, which intensified the perception of one’s own and others’ suffering, facilitating more compassionate attitudes; (c) self-compassion exercises, based on the work of Dr. Kristin Neff [16], with the aim of fostering an attitude of kindness and understanding towards oneself in times of difficulty rather than resorting to self-criticism; (d) guided imagery, which involved visualizing situations in which compassion was given or received, thus reinforcing compassionate attitudes applicable in real situations; (e) gratitude journaling, where students recorded experiences and reflections related to acts of kindness and gratitude, promoting recognition of daily compassionate interactions; and (f) active listening and empathy, a technique that fostered deep understanding of others’ emotions and perspectives without judgment, key to the development of compassion.

At the end of each session, autonomous exercises were assigned to be performed ideally on a daily basis, with a minimum commitment of between 15 and 30 min, although students could repeat them several times a day according to their needs. It is important to note that the performance of these exercises was considered desirable but not mandatory, as no coercive strategies were implemented to ensure their daily practice.

### 2.3. Instruments

The measurement of self-compassion in this study was performed using the Self-Compassion Scale (SCS-26), a self-administered instrument developed by Kristin Neff in 2003. We used the version translated into Spanish by García-Campayo [21], validated in Chile by Araya-Véliz in 2017 [22]. This scale consists of 26 items, assessed using a 5-point Likert scale ranging from 1 (almost never) to 5 (almost always), and it is organized into six subscales, namely, self-kindness, self-judgment, shared humanity, isolation, mindfulness, and overidentification, with specific items for each. The higher the score, the higher the level in each subdimension. The reliability of the subscales has been reported to have reliability coefficients ranging from 0.66 to 0.78.

The Perceived Stress Scale (PSS-14) was used [23]. It is a self-report instrument oriented toward measuring the level of stress perceived during the last month as a single measure. The instrument consists of 14 items with a 5-point Likert scale format (0 = never, 1 = almost never, 2 = occasionally, 3 = often, 4 = very often), with acceptable reliability evidence (α = 0.79) [6].

The State-Trait Anxiety scale [5] was used. This instrument evaluates State Anxiety (A-E) (i.e., the transitory emotional condition of a person), wherein Trait Anxiety (A-R) is understood as a permanent emotional condition of tension [5], accounting for a relatively permanent characteristic of a person related to their tendency to respond with a high degree of anxiety to situations perceived as threatening. The adaptation to the Chilean population showed an internal consistency of 0.92 for the A-E scale and 0.87 for A-R [24].

### 2.4. Ethical Considerations

This study obtained the approval of the Ethics and Scientific Research Committee of the Universidad de Antofagasta (402/2022) and complied with the principles of the Declaration of Helsinki. Students were informed of the voluntary nature of this study, and it was made clear to them that they could withdraw at any time. During the first session, they were asked to sign an informed consent form.

The confidentiality and anonymity of the participants were also guaranteed, in accordance with national regulations on personal data protection. To preserve anonymity, participants were asked to create a personal code to be used in both the pretest and the posttest. In order to reduce the risk of forgetting this code, it was suggested that they use the last four digits of their personal identification number. This measure allowed anonymous tracking of the data throughout the study, ensuring the protection of each participant’s personal information.

### 2.5. Data Collection

Data collection was carried out by means of physical surveys self-administered in person one week before (T1) the beginning of the intervention (T2) and one week after its completion (T3). During these sessions, students completed questionnaires that included sociodemographic data and the previously described scales to measure the variables of interest of this study.

### 2.6. Data Analysis

In the first analysis, data were presented as means and standard deviations. The Kolmogorov–Smirnov test was used to evaluate the distribution of the variables. This analysis indicated that all scaling variables had a non-normal distribution. Consequently, the Wilcoxon signed-rank test was used to analyze changes before and after the intervention. The results were considered statistically significant, with a *p*-value less than 0.05.

In addition, G*Power software version 3.1 [25] was used to calculate effect size (Cohen’s *d*) and statistical power of median differences. The effect size was interpreted through Cohen’s *d*, as a function of the magnitude of the observed changes. A value of 0.2 indicates a small effect size, suggesting that the change or difference detected is relatively subtle. A value of 0.5 represents a medium effect size, indicating a change of moderate magnitude, while a value of 0.8 or greater corresponds to a large effect size, implying a substantial difference in the results [26].

Subsequently, two path analyses were conducted to assess the connections between minutes of autonomous practice (T2), self-compassion (T3), stress (T3), and anxiety, both in its state and trait forms (T3). In the first model (M1), we examined how minutes of autonomous practice (T2) influenced levels of stress (T3), state anxiety (T3), and trait anxiety (T3), controlling for gender, age, stress (T1), state anxiety (T1), and trait anxiety (T1) before the intervention. In the second model (M2), a mediation analysis was conducted to assess whether post-intervention self-compassion (T3) mediated the relationship between minutes of autonomous practice (T2) and stress (T3), state anxiety (T3), and trait anxiety (T3), again controlling for pre-intervention sex, age, self-compassion (T1), stress (T1), state anxiety (T1), and trait anxiety (T1).

Model fit was assessed using Chi-square (χ^2^), root mean square error of approximation (RMSEA), comparative fit index (CFI), and Tuker-Lewis index (TLI) statistics. According to the criteria developed by Schreiber [27], an RMSEA ≤ 0.08, a CFI ≥ 0.95, and a TLI ≥ 0.95 are considered indicators of a good fit. The robust maximum likelihood (MLR) method, appropriate for data without multivariate normality, was employed [28]. Analyses were performed using SPSS version 25 [29] and Mplus version 8.2 [28].

To evaluate the power of the mediation model, Hayes’ [30] approach was considered, which posits that since *Y* depends exclusively on *X* and *M*, the direct and indirect effects of *X* on *Y* remain constant, regardless of whether they are estimated together with other variables in the model or independently. Based on this principle, a power analysis for indirect effects was conducted using Monte Carlo simulations [31], covering all three possible result pathways to ensure an accurate assessment of the model’s statistical power.

## 3. Results

### 3.1. Pre–Post Intervention Analysis

Most of the students were female (82.2%), and their ages ranged from 18 to 27 years old, with a mean of 19.3 (SD = 1.7). Data analysis revealed significant improvements in the levels of perceived stress, state anxiety, and trait anxiety after the intervention. For perceived stress, the mean decreased from 33.13 (SD = 8.21) before the test to 21.8 (SD = 12.93) after the test, with a statistically significant difference (Z = −4.312, *p* = 0.000). The effect size (Cohen’s *d* = 0.83) indicates a large effect, and the statistical power (1 − β = 0.99) suggests a high probability of detecting a true change. In the state anxiety dimension, a significant decrease was also observed, falling from a mean anxiety of 31.53 (SD = 11.7) before the test to 16.9 (SD = 17.53) after the test. The analysis showed a significant difference (Z = −4.234, *p* = 0.000) and a large effect size (Cohen’s *d* = 0.74), with an equally high statistical power (1 − β = 0.99). Finally, in the case of trait anxiety, although there was a decrease in the mean from 35.24 (SD = 10.6) to 29.3 (SD = 14.17) after the intervention, the effect size was moderate (Cohen’s *d* = 0.34), with a statistical power of 0.72. Nevertheless, the difference remained statistically significant (Z = −4.603, *p* = 0.000) (Table 1).

### 3.2. Mediation Model

In the first model (M1), the impact of minutes of autonomous practice (T2) on stress (T3), state anxiety (T3), and trait anxiety (T3) was evaluated, controlling for the variables sex, age, stress (T1), state anxiety (T1), and trait anxiety (T1). Model goodness-of-fit indices (CFI = 1.000; TLI = 1.000; RMSEA = 0.000) were consistent.

**Figure 1:**
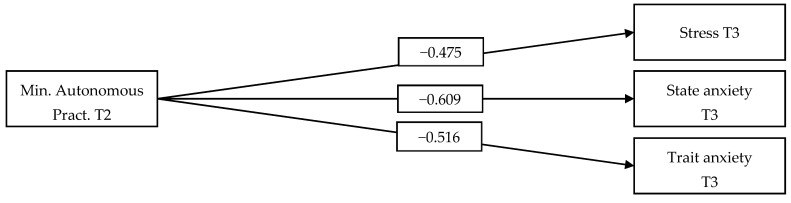
Effect of Minutes of Autonomous Practice on Stress, State Anxiety and Trait Anxiety.

As shown in Figure 1 (M1), minutes of autonomous practice exhibited a statistically significant, inverse, moderate-magnitude relationship with stress (T3) (β > 0.30). In addition, an inverse relationship with a large magnitude was observed with respect to state anxiety (T3) and trait anxiety (T3; β > 0.50).

The relationship between minutes of autonomous practice (T2) and stress, trait anxiety, and state anxiety (T3) is depicted above. Note: In this analysis, we controlled for the effects of pre-intervention stress, state anxiety, and trait anxiety (T1). Solid trajectories indicate significant effects on relationships (*p* < 0.05).

After having estimated the direct relationship between minutes of autonomous practice (T2) and stress (T3), state anxiety (T3), and trait anxiety (T3) in the first model (M1), we proceeded with the mediation analysis (M2). In this second model, self-compassion (T3) was included as a mediating variable between minutes of autonomous practice (T2) and the variables of stress (T3), state anxiety (T3), and trait anxiety (T3), controlling for sex, age, self-compassion (T1), stress (T1), state anxiety (T1), and trait anxiety (T1).

**Figure 2:**
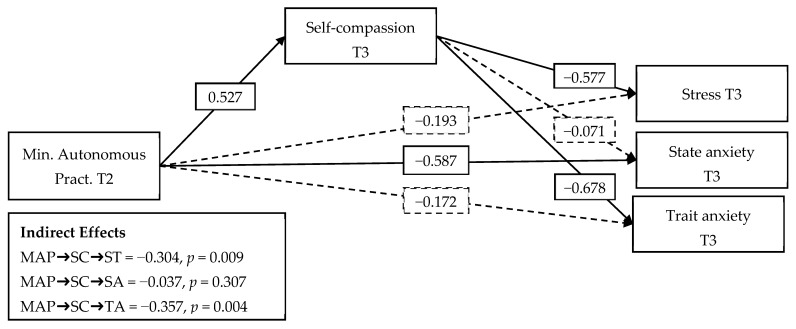
Moderating Effect of Self-Compassion on the Relationship Between Minutes of Autonomous Practice and Stress, State Anxiety, and Trait Anxiety. Note: MAP = minutes of autonomous practice; SC = self-compassion; ST = stress; SA = state anxiety; TA = trait anxiety. The analysis controlled for the effects of sex, age, self-compassion, stress, and pre-intervention state and trait anxiety (T1). Solid trajectories indicate significant relationships (*p* < 0.05). Non-significant paths are represented with a dashed line.

The mediation model (M2) showed adequate goodness-of-fit indicators (CFI = 1.000; TLI = 1.000; RMSEA = 0.000), according to the criteria recommended by Schreiber [30], suggesting that the model correctly represents the observed relationships. As shown in Figure 2, self-compassion fully mediated the relationship between minutes of autonomous practice and stress (indirect effect = −0.304, *p* = 0.009), as well as the relationship between minutes of autonomous practice and trait anxiety (indirect effect = −0.357, *p* = 0.004).

We did not find a mediating effect of self-compassion on the relationship between minutes of autonomous practice and state anxiety (indirect effect = −0.037, *p* = 0.307). However, an inverse direct effect, large in magnitude and statistically significant, on state anxiety was observed (β = −0.587, *p* < 0.000).

Shown above are the results of a mediation analysis of self-compassion levels in the relationship between minutes of autonomous practice and stress, trait anxiety, and state anxiety (T3). Note: In this analysis, we controlled for the effects of gender, age, self-compassion, pre-intervention (T1) stress, and state and trait anxiety. Solid paths indicate significant effects on relationships (*p* < 0.05). Non-significant paths are shown with a dashed line.

The sample of 45 participants was adequate for detecting statistically significant effects of considerable magnitude (β > 0.50). The analysis revealed a power of 1 − β = 0.96 for the pathway from “Minimum Autonomous Practice T2 (SD = 141.307)” through “Self-compassion (SD = 1.031)” to “Stress T3 (SD = 12.931)”, and a power of 1 − β = 0.97 for the pathway to “Trait Anxiety T3 (SD = 14.170)”. These results suggest that the sample size was also sufficient to identify smaller effects in these specific cases. However, the power was insufficient (1 − β = 0.08) to detect small effects in the pathway to “State Anxiety T3 (SD = 17.535)”, indicating a limitation in detecting subtle effects in this last case.

## 4. Discussion

The results of this study confirm the effectiveness of compassion training in reducing levels of perceived stress and anxiety (trait and state) in nursing students. The intervention showed significant improvements in all the dimensions assessed, with large effect sizes with respect to perceived stress and state anxiety and a moderate effect on trait anxiety. These findings are consistent with previous research suggesting that compassion and its components may serve as effective strategies for stress and anxiety management in health discipline students [1,15].

Regarding perceived stress, the remarkable decrease observed, with a large effect size, reinforces the hypothesis that compassion training provides students with tools to cope with stressful situations more effectively. This reduction is relevant not only to their emotional well-being but also to their ability to cope with academic and clinical demands, as suggested by previous studies [2]. The significant impact of the intervention on state anxiety status (Cohen’s *d* = 0.74) also supports the literature indicating that compassion practice has an immediate effect on emotional regulation, improving responses in the face of acute stressful situations [18].

On the other hand, although trait anxiety also decreased significantly, the corresponding moderate effect size (Cohen’s *d* = 0.34) suggests that changes in this dimension are more subtle and may require a more prolonged or intensive approach to allow greater improvements to be observed. This finding is consistent with the literature, which indicates that personality traits, such as trait anxiety, may be more resistant to change than state anxiety, for which there is a better response to timely interventions [32].

Mediation analysis revealed that the effects of time spent in compassion training on reducing stress and trait anxiety were mediated by self-compassion. This suggests that students who practiced compassionate techniques longer experienced greater increases in their levels of self-compassion, which in turn contributed to reductions in their levels of stress and trait anxiety. This finding supports theories positing that self-compassion is a key factor in emotional regulation and improving well-being [16]. In contrast, the state was not mediated by self-compassion, which could be explained by the more momentary and fluctuating nature of this type of anxiety compared to trait anxiety, which has more stable components [1].

In particular, the results obtained have direct implications for health education. Incorporating subjects that include compassion training may not only reduce the emotional distress of students but also prepare future professionals to face the emotional demands of clinical practice. These programs could contribute to the creation of educational environments that are more humanized and aligned with the promotion of the mental health and well-being of students

### Limitations and Future Lines of Research

A limitation of this study is the lack of a control group, which prevents us from ruling out other external factors that could have influenced the observed results. Future research could consider the use of randomized controlled designs to strengthen the validity of the findings. In addition, we suggest investigating the long-term effects of these interventions, since the impact of compassion training may increase with time and continued practice [15]. It would also be relevant to explore how compassion towards others and compassion received influence students’ emotional well-being, as these factors were not addressed in this study.

It is also important to note that the second measurement was taken at the end of the academic semester, which may also be related to lower levels of stress and anxiety.

## 5. Conclusions

This study provides evidence that compassion training is an effective intervention for reducing perceived stress and anxiety in nursing students, highlighting it as a valuable strategy for enhancing their emotional regulation skills and preparing them for the emotional challenges inherent in professional practice within the healthcare field. The findings underscore the importance of including compassion-based approaches in educational programs, not only to promote students’ emotional well-being but also to equip them with tools to handle the emotional demands of clinical practice. Additionally, this study contributes new insights to the potential of compassion training, showing that increases in self-compassion levels mediated the relationship between autonomous practice time and the reduction in stress and trait anxiety. These results emphasize the need to integrate compassion into the curricula of health disciplines as a preventive and supportive approach to enhance both the personal well-being of future professionals and their readiness in order to facilitate a more humanized professional practice.

For future studies, we suggest implementing a randomized, controlled design to enhance the validity of the findings, incorporating a control group to rule out the influence of external factors on the observed results. Additionally, it would be valuable to investigate the long-term effects of compassion training, as its impact may intensify over time with continued practice. Exploring how compassion towards others and compassion received influence students’ emotional well-being would also be relevant, as these aspects were not addressed in the present study.

Another recommendation is to assess stress and anxiety levels at different points throughout the academic year to analyze the potential effect of the academic context on the results, such as increased demands toward the end of the semester. Finally, future studies could compare the effects of compassion training among students from different health disciplines, contributing to a more comprehensive understanding of the applicability of this intervention within health education.

## Figures and Tables

**Table 1:** Pre–post intervention comparison of levels of perceived stress, trait anxiety, and state anxiety.

	Pre-Test (*n* = 45)		Post-Test (*n* = 45)		Z	*p*	Cohen’s *d*	1 − β
	M	SD	M	SD				
Perceived Stress	33.13	8.21	21.8	12.93	−4.312	0.000 *	0.83	0.99
Anxiety Status	31.53	11.7	16.9	17.53	−4.234	0.000 *	0.74	0.99
Trait Anxiety	35.24	10.6	29.3	14.17	−4.603	0.000 *	0.34	0.72

Note: M = mean; SD = standard deviation; Z = Wilcoxon test; *p* = significance level; Cohen’s *d* = effect size; β = statistical power. * *p* ≤ 0.05.

## Data Availability

The data supporting the results reported in this study can be made available upon request. Please contact the corresponding author for access to the data.

## Public Involvement Statement

There was no public involvement in any aspect of this research.

## Guidelines and Standards Statement

This study followed the STROBE checklist. A complete list of reporting guidelines can be accessed via the equator network: https://www.equator-network.org/ (accessed on 29 September 2024).
